# Rapid replacement by non-vaccine pneumococcal serotypes may mitigate the impact of the pneumococcal conjugate vaccine on nasopharyngeal bacterial ecology

**DOI:** 10.1038/s41598-017-08717-0

**Published:** 2017-08-15

**Authors:** Brenda Kwambana-Adams, Blake Hanson, Archibald Worwui, Schadrac Agbla, Ebenezer Foster-Nyarko, Fatima Ceesay, Chinelo Ebruke, Uzochukwu Egere, Yanjiao Zhou, Maze Ndukum, Erica Sodergren, Michael Barer, Richard Adegbola, George Weinstock, Martin Antonio

**Affiliations:** 1 Vaccines and Immunity Theme, Medical Research Council Unit The Gambia (MRCG), Fajara, The Gambia; 2 The Jackson Laboratory for Genomic Medicine, Farmington, CT USA; 3 London School of Hygiene and Tropical Medicine, London, UK; 4 The Genome Instituted (Washington University in St Louis), St. Louis, Missouri USA; 5 Infection, Immunity and Inflammation, University of Leicester, Leicester, UK; 6 GSK, Wavre, Belgium; 7 Division of Microbiology & Immunity, Warwick Medical School, University Of Warwick, Coventry, UK

## Abstract

There is growing concern that interventions that alter microbial ecology can adversely affect health. We characterised the impact of the seven-valent pneumococcal conjugate vaccine (PCV7) on pneumococcal carriage and the bacterial component of the nasopharyngeal microbiome during infancy. Newborns were recruited into three groups as follows: Group1 (n = 33) was the control group and comprised infants who received PCV7 after 6 months and came from unvaccinated communities. Group 2 (n = 30) came from unvaccinated communities and Group 3 (n = 39) came from vaccinated communities. Both group 2 and 3 received PCV7 at 2, 3 and 4 months. Culture and 16 S rRNA gene sequencing were performed on nasopharyngeal specimens collected at regular intervals from infants. Nasopharyngeal carriage of PCV7 serotypes in Group 1 was significantly higher than in Group 2 and 3 (p < 0.01). However, pneumococcal carriage remained comparable due to an expansion of non-vaccine serotypes in Groups 2 and 3. Determination of phylogenetic dis(similarities) showed that the bacterial community structures were comparable across groups. A mixed effects model showed no difference in community richness (p = 0.15) and Shannon **α**-diversity (p = 0.48) across the groups. Immediate replacement of pneumococcal vaccine serotypes with non-vaccine serotypes may mitigate the impact of PCV7 on nasopharyngeal bacterial community structure and ecology.

## Introduction

*Streptococcus pneumoniae*, the pneumococcus, causes several serious and often life-threatening infections including meningitis, pneumonia and bacteraemia^1^. The 7-valent pneumococcal polysaccharide-diphtheria CRM_197_ protein conjugate vaccine (PCV7) was licensed in 2000 and markedly reduced the carriage of vaccine serotypes^2, 3^ and the incidence of invasive disease^4–6^. Childhood deaths attributed to pneumococcus reduced from 735,000 in 2000 to 476,000 in 2008 following widespread use of PCVs. The 10-valent PCV formulation (PCV10) includes all the serotypes in PCV7 and serotypes 1, 5 and 7F^7, 8^. The 13-valent PCV (PCV13) formulation includes all the additional serotypes 3, 6A and 19A. PCV10 and PCV13 have replaced PCV7 and had been introduced in 132 countries globally as of September 2016^7^.

Although PCVs are a remarkable public health success, their long-term utility is threatened by the emergence of serotype replacement and possibly species replacement. Although serotype replacement^9, 10^ and serotype switching^11–13^ associated with widespread use of PCVs have been reported, the impact of vaccination on the nasopharyngeal microbial ecology, which may influence replacement disease, has not been determined among West African children. Few studies have reported changes in the epidemiology of bacterial diseases associated with widespread use of PCV7^14–16^; however, it is unclear how these changes are related to nasopharyngeal microbial colonisation. The nasopharynx is an important reservoir of commensal and pathogenic microbes, which can migrate to the sinuses, middle ear and lower respiratory tract and invade the blood system. The issue of replacement in carriage and disease is of particular interest within the Gambian context where infant pneumococcal nasopharyngeal carriage rates exceed 90% and PCV7 protects against approximately 63% of previously circulating serotypes^17, 18^. PCV7 was introduced in the routine immunization programme in The Gambia in August 2009, and was replaced by PCV13 in June 2011.

We set out to determine the effects of PCV7 on nasopharyngeal microbial ecology and assess its wider public health impact by asking whether vaccination significantly alters the composition and structure of the infant nasopharyngeal microbiome. A birth cohort of 102 Gambian newborns recruited into three groups with distinct PCV7 vaccine exposures was rigorously sampled for one year to address this question.

## Results

### Characteristics of study participants

**Figure 1:**
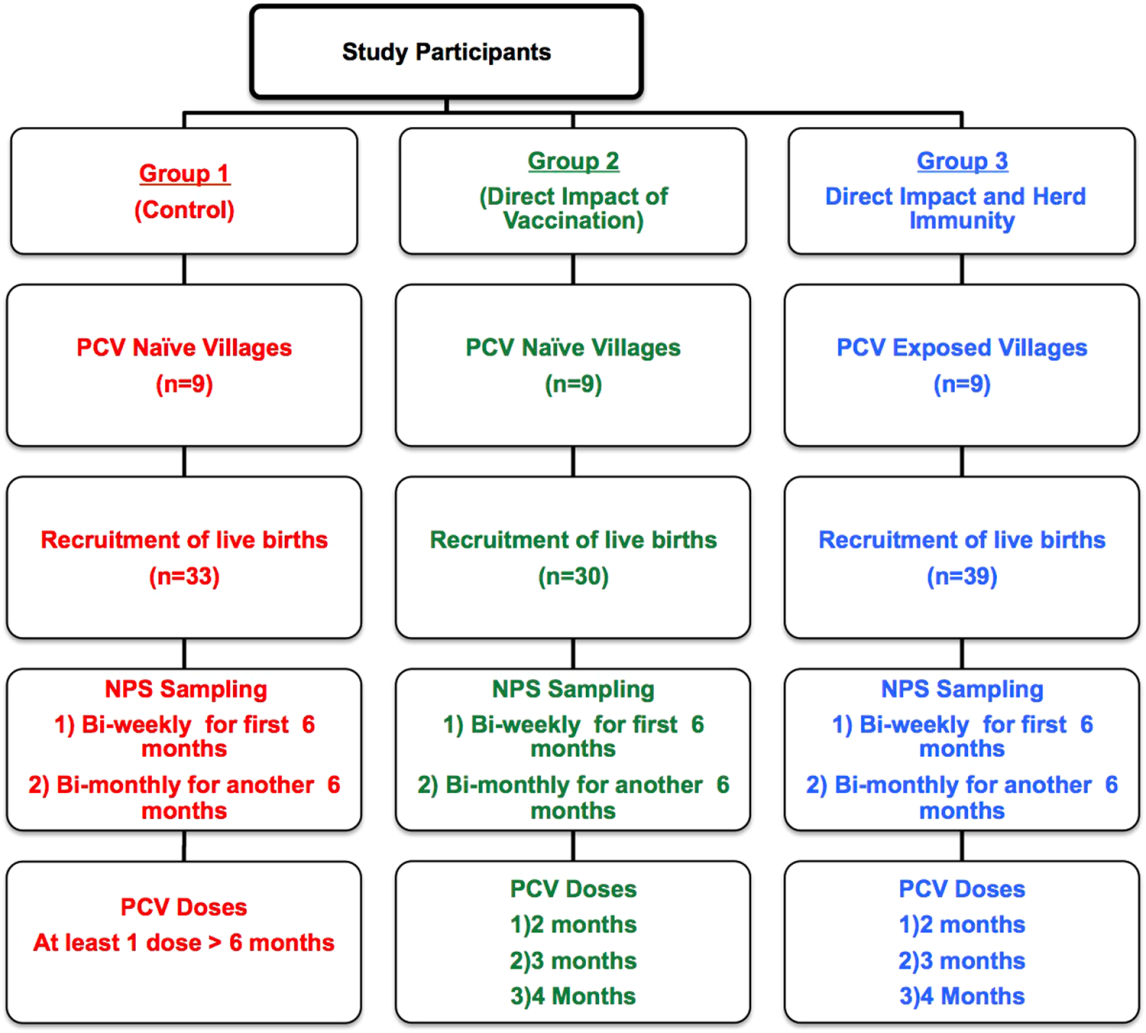
Flowchart of infant recruitment and sample collection.

All newborns from participating villages for whom informed parental consent was obtained were recruited into the study until each of the vaccination groups had at least 30 children. A total of 102 children were recruited into each of the study groups as follows, Group 1 (n = 33), Group 2(n = 30) and Group 3 (n = 39). The baseline characteristics of the newborns recruited into in each of the study groups are summarised in Table 1. There were no significant differences in the baseline characteristics across the study groups except for birthplace, maternal vaccination and sibling vaccination status. Group 3 newborns came from villages with widespread PCV7 vaccination and were expected to have high rates of maternal and sibling vaccination. The study completion rate was 94% (96/102); 6 children did not complete the study, 3 died and 3 dropped out or were lost to follow-up. None of the children that died had been vaccinated with PCV7. 1595 nasopharyngeal swabs were collected from the children, with a mean of 16 specimens (range 1 to 17) per infant over the 12 months of follow-up as outlined in Fig. 1.

**Table 1:** Baseline characteristics of newborns and their mothers by study group.

Characteristics	Category	Group 1 (n = 33)	Group 2 (n = 30)	Group 3 (n = 39)	p-value
Sex, n (%)	Male	20 (60.6)	13 (43.3)	19 (48.7)	0.37^a^
	Female	13 (39.4)	17 (56.7)	20 (51.3)	
Birthplace, n (%)	Home	9 (27.3)	20 (66.7)	23 (59.0)	<0.01^b^
	Health Centre	4 (12.1)	2 (6.7)	10 (25.6)	
	Hospital	20 (60.6)	8 (26.7)	6 (15.4)	
Birth type, n (%)	Vaginal	33 (100)	30 (100)	39 (100)	N/A
Mother’s age in years, n (%)	<20	3 (9.1)	2 (6.7)	6 (15.4)	0.84^b^
	20–34	20 (60.6)	20 (66.7)	23 (59.0)	
	≥35	10 (30.3)	8 (26.7)	10 (25.6)	
Gestation, n (%)	Full term	33 (100)	30 (100)	38 (97.4)	0.99^b^
	Premature	0 (0.0)	0 (0.0)	1 (2.6)	
Siblings, n (%)	0	4 (12.1)	4 (13.3)	6 (15.4)	0.75^b^
	1–3	16 (48.5)	12 (40.0)	21 (53.9)	
	>3	13 (39.4)	14 (46.7)	12 (30.8)	
Ethnicity, n (%)	Mandinka	6 (18.2)	7 (23.3)	12 (30.8)	0.38^a^
	Jola	18 (54.6)	18 (60.0)	23 (59.0)	
	Other	9 (27.3)	5 (16.7)	4 (10.3)	
Birth weight in kg*	Median (min, max)	3.1 (2.5–3.9)	3 (2.6–3.4)	3 (1.9–4.3)	0.34^c^
Mother vaccinated, n (%)	No	33 (100)	30 (100)	18 (46.2)	<0.01^a^
	Yes	0 (0.0)	0 (0.0)	21 (53.8)	
Vaccinated siblings, n (%)	No	33 (100)	30 (100)	34 (87.2)	0.01^b^
	Yes	0 (0.0)	0 (0.0)	5 (12.8)	

^a^Chi square test. ^b^Fisher’s exact test. ^c^Kruskal-Wallis test.

^*^Birth weight was not recorded for 15, 16 and 11 of Group 1, 2 and 3 newborns respectively.

### Pneumococcal carriage dynamics

**Figure 2:**
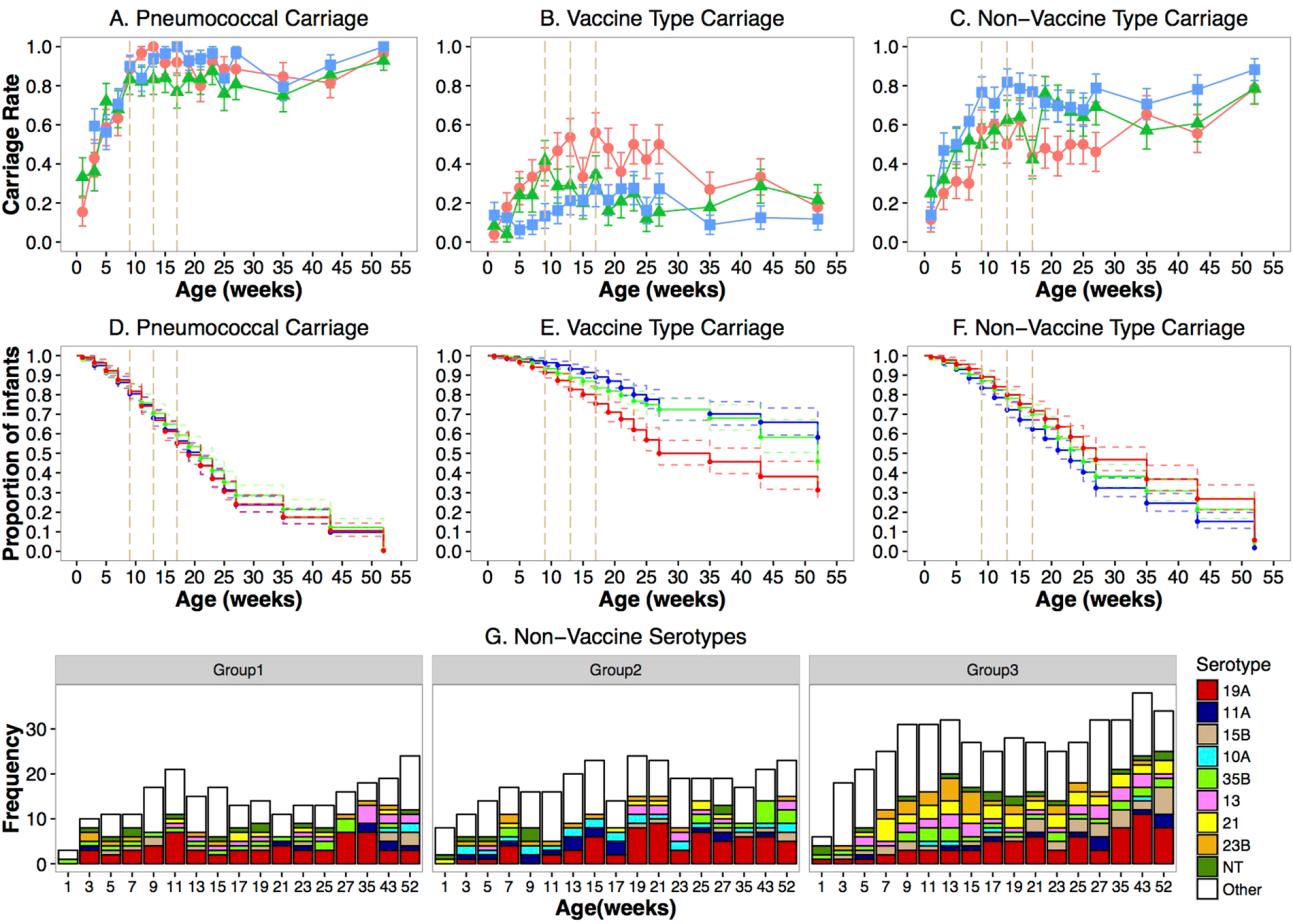
Nasopharyngeal carriage dynamics of pneumococcus, vaccine and non-vaccine serotypes in the first year of life. Carriage rates of pneumococcus (**A**), vaccine (**B**) and non-vaccine serotypes (**C**). Kaplan Meier Survival Curve for time to first acquisition of pneumococcus (**D**), vaccine serotypes (**E**) and non-vaccine serotypes (**F**). Distribution of the top ten vaccine serotypes by group (**G**). The vertical dashed lines are the study 2, 3, and 4 month vaccination time points. P values generated from an adjusted logistic regression model. The line colour denotes the group: red for Group 1, green for group 2 and blue for Group 3.

Pneumococcus was found in 79% (1258/1595) of the nasopharyngeal specimens by culture. Vaccine serotypes were detected in 30% (383/1258) of the pneumococcus positive specimens by the sweep latex agglutination serotyping method^19^. Pneumococcal carriage among Group 1 infants in the first 12 months of life was 79%(95CI: 76–83), comparable to Group 2 75%(95% CI: 71–79), p = 0.28 and Group 3 82%(95% CI: 79–85), p = 0.29. In contrast, carriage of PCV7 serotypes was 37% (95% CI: 32–41) among Group 1 infants, significantly higher than among Group 2 infants 20% (95% CI: 17–24%), p < 0.01 and Group 3 infants 17% (95CI: 14–20), p < 0.01. Pneumococcal, vaccine type and non-vaccine type carriage across the 12 months of follow-up are shown in Fig. 2A,B and C. The carriage of vaccine type pneumococci decreased by nearly half following the first dose of PCV7 given at 9 weeks among Group 2 infants (Fig. 2B). Vaccine type carriage declined between 35 and 52 weeks among Group 1 infants following the nationwide introduction of PCV7. Although pneumococcal acquisition was similar across all three groups (Fig. 2D), Group 1 infants acquired vaccine type pneumococci more rapidly than Group 3 infants (Fig. 2E). The most common non-vaccine serotypes across all the vaccination groups were 19A, 23B and 35B. Carriage of serotype 15B was most common among Group 3 infants who came from heavily vaccinated villages and received PCV at 2, 3 and 4 months (Fig. 2G).

Carriage of pneumococcus among children born to vaccinated mothers was 78% (95% CI: 76–81%), comparable to carriage rates among infants born to unvaccinated mothers 83% (95% CI: 78–87%), p < 0.065. However, infants with vaccinated mothers had significantly lower carriage of pneumococcal vaccine serotypes, 19%(95% CI:14–23%) compared to infants with unvaccinated mothers 25%(95% CI: 23–28%), p = 0.014. Nonetheless, there was no significant difference in carriage of vaccine serotypes among children with at least one vaccinated sibling 23%(95% CI: 14–32%), and those without any vaccinated siblings 24%(95% CI: 22–26%), p = 920.920. Infants born at a health facility (health center or hospital) had significantly higher carriage of pneumococcal vaccine serotypes 27%(95% CI: 23–30%) than infants born at home 21%(95% CI: 19–24%), p = 0.009. There was no difference in the carriage of pneumococcus among children born at health facilities 79%(95% CI: 76–82%) and at home 79%(95% CI: 77–82%), p = 0.862.

### Nasopharyngeal microbial communities

**Figure 3:**
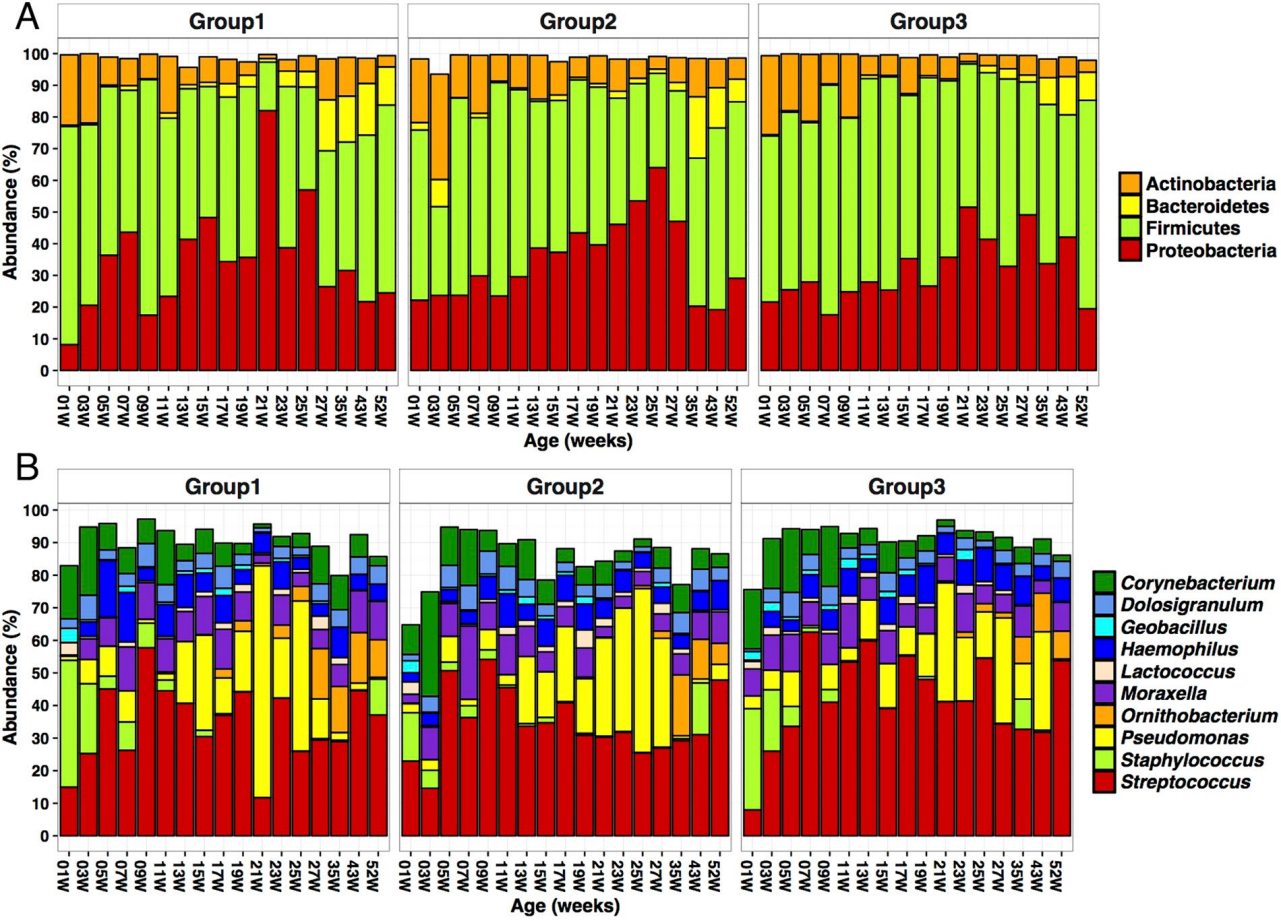
Composition of the nasopharyngeal microbiome in the first year of life. The distribution of the top 5 phyla (**A**) and the top 10 genera (**B**) in the nasopharyngeal microbiome from birth to 52 weeks for each vaccination group is shown.

Of the 1595 nasopharyngeal specimens collected, 16S rDNA deep sequencing was successfully performed on 91% (1460/1595). Initial amplification and/or sequencing with a minimum of 500 reads per sample failed in 9% of (135/1595) NPS specimens. The sequences from this dataset were binned into 814 operational taxonomic units (OTUs) representing at least 297 genera and 15 Phyla. Firmicutes, Proteobacteria, Bacteroidetes and Actinobacteria accounted for at least 90% of the nasopharyngeal microbiota across all time points in each of the groups (Fig. 3A). Likewise, the top ten genera, which included *Streptococcus*, *Staphylococcus*, *Corynebacterium*, *Pseudomonas*, *Moraxella* and *Haemophilus*, accounted for more than 70% of the microbiota throughout the first 12 months of life (Fig. 3B). These microbes form the backbone of the infant nasopharyngeal microbiome while there are numerous low abundance microbes, which may account for most of the diversity within and across individuals.

In the first week of life, *Staphylococcus*, *Corynebacterium* and *Streptococcus* were the dominant genera (Fig. 3B). However, while the initial dominance of *Staphylococcus* decreased in the subsequent weeks, the relative abundance of *Streptococcus* and *Pseudomonas* increased, accounting for over 50% of the microbiota at some visits.

### Effect of vaccination on community composition and structure

**Figure 4:**
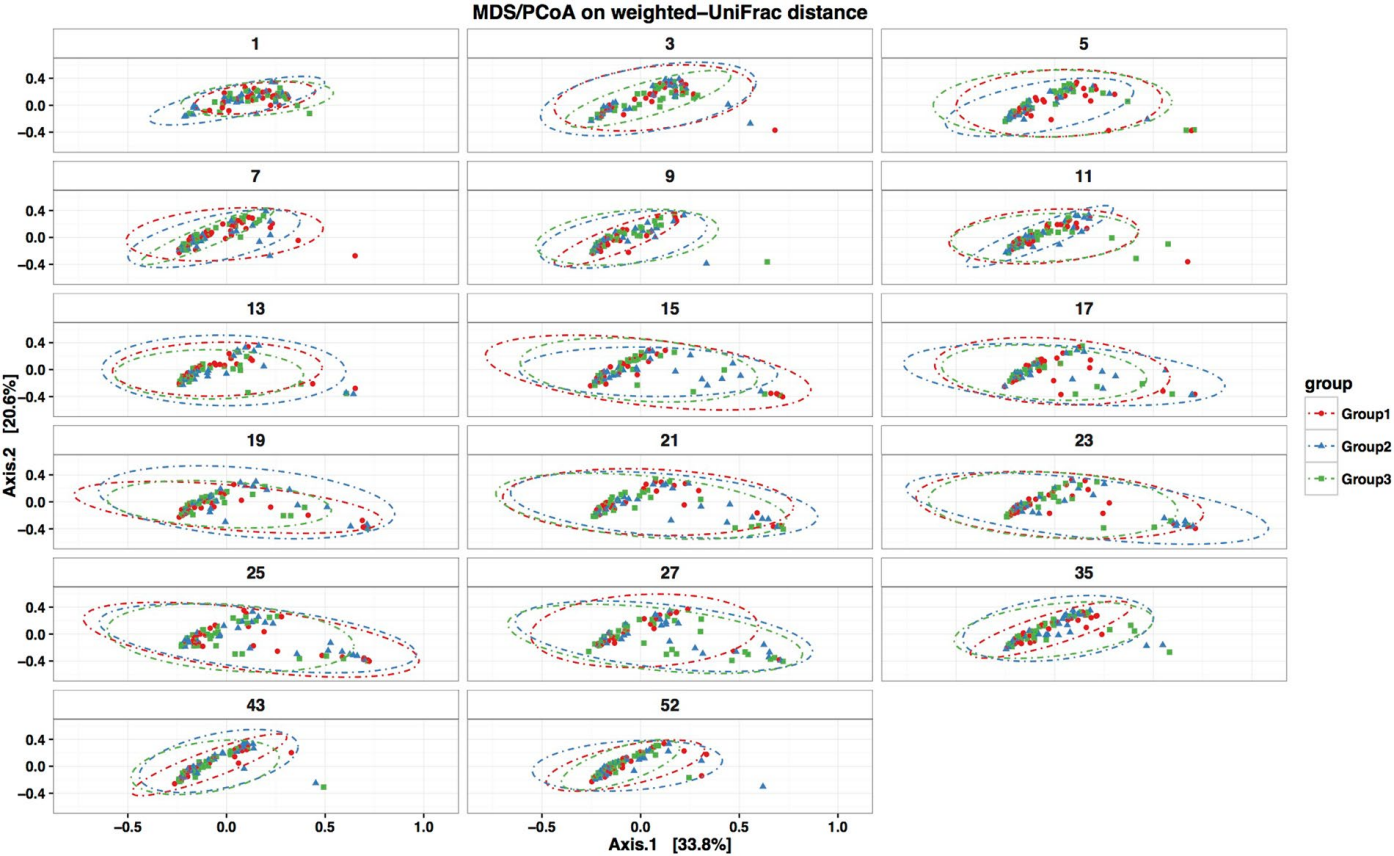
Clustering of nasopharyngeal microbial profiles by group in the first year of life. The phylogenetic dis(similarities) among communities from the three vaccination groups were evaluated by MDS/PCoA on weighted-UniFrac distances. The dotted eclipses show the 95% confidence intervals for the ordinations for each group at each time point. Each circle represents the microbial profile of a nasopharyngeal specimen from an infant and the colour denotes the vaccination group; red for Group 1, green for group 2 and blue for Group 3. The distance between circles indicates the variability between nasopharyngeal microbial profiles i.e. longer distances show greater dissimilarity.

To evaluate the effect of PCV7 vaccination on overall microbial composition, the phylogenetic dis(similarities) among communities from the three groups were evaluated by MDS/PCoA on weighted-UniFrac distance. There were no distinct shifts in community composition associated with vaccination group as communities from all three groups clustered closely together at each of the 17 age-based visits (Fig. 4). The distribution of the most abundant OTUs across time among the groups appeared very similar at both Phylum and Genus taxonomic levels (Fig. 3). MDS/PCoA on weighted-UniFrac suggested higher variability in community composition between the 15^th^ and 27^th^ weeks after birth compared to the early and latter weeks (Fig. 4).

**Figure 5:**
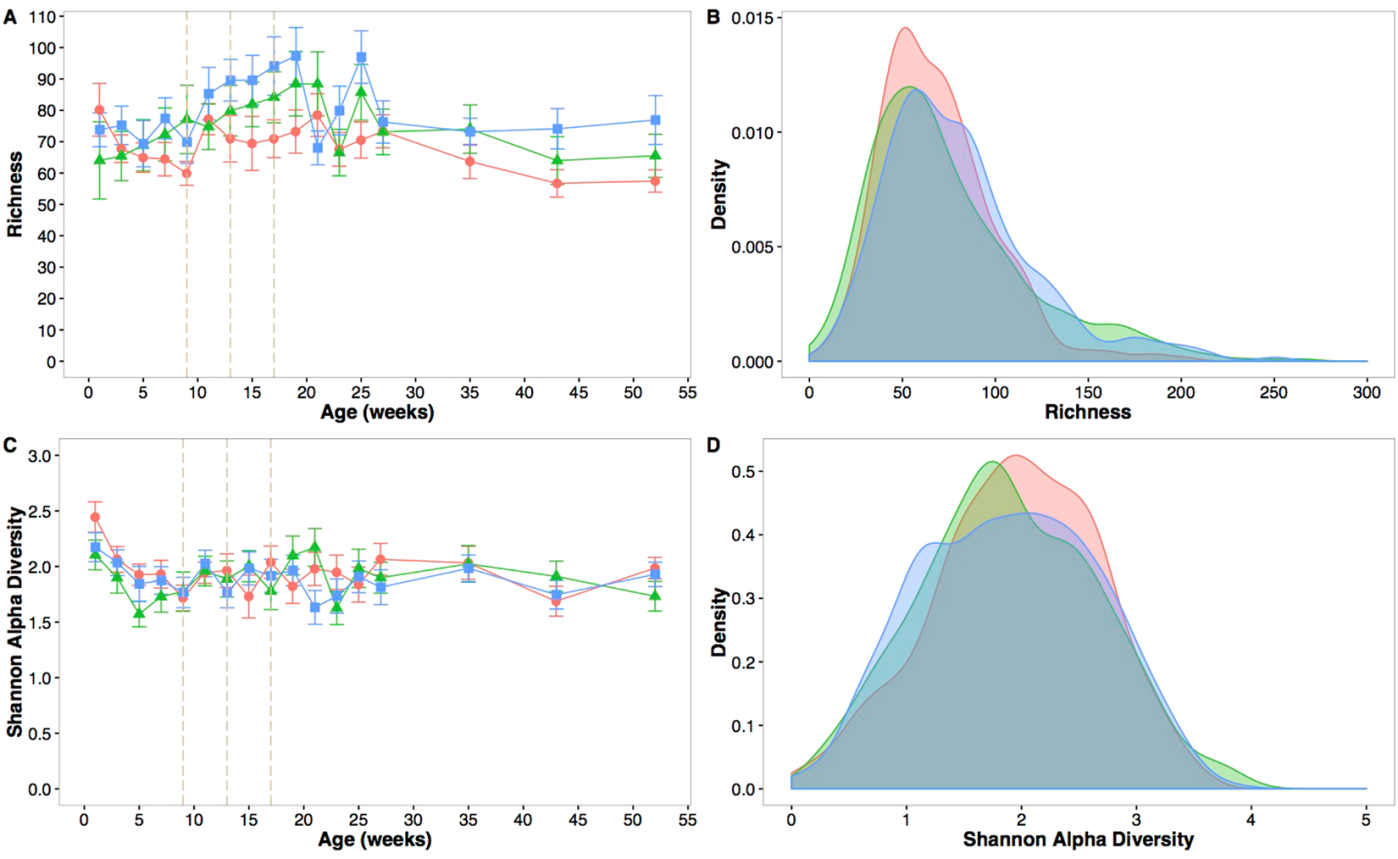
Nasopharyngeal microbiome richness and Shannon α-diversity in the first year of life by group. Richness over time (**A**); Density plot of richness (**B**); Shannon α-diversity in the first year of life (**C**); Shannon α-diversity in the first year of life (**D**). The vertical dashed lines are the study vaccination time points. The colour denotes the vaccination group; red for Group 1, green for group 2 and blue for Group.

A random intercept mixed-effects model was used to investigate the interaction between richness (Supplementary Table 1) and Shannon **α**-diversity (Supplementary Table 2) with vaccination group and age adjusting for time-variant and time-invariant explanatory variables. The square root of richness was higher between 11 and 17 weeks after birth (adjusted difference: 0.75 (95% CI: 0.41, 1.10) and between 19 and 27 weeks after birth (adjusted difference: 0.82 (95% CI: 0.30, 1.34) compared to the first 9 weeks of life. No difference in richness was found between the first 9 weeks of life and the last 20 weeks of the first year. These trends are visualized in Fig. 5A. Nasopharyngeal microbial communities harboured a median of 67 (range 1–264) OTUs per specimen and the distribution of richness was similar across all three groups (Fig. 5B). Shannon **α**-diversity decreased over time during the first 9 weeks of life (adjusted time effect: −0.06 (95% CI: −0.08, −0.03)) and became steady thereafter (visualized in Fig. 5C). The distribution of Shannon **α**-diversity was similar across all three groups (Fig. 5D). There was no evidence of difference in richness (p = 0.15) and Shannon **α**-diversity across the three vaccination groups (p = 0.48) in the mixed effects random intercept model. Other factors such as breastfeeding and mother’s age appeared to have significant associations with both richness and Shannon **α**-diversity (Supplementary Tables 1 and 2).

### Effect of PCV7 vaccination on individual OTUs

**Figure 6:**
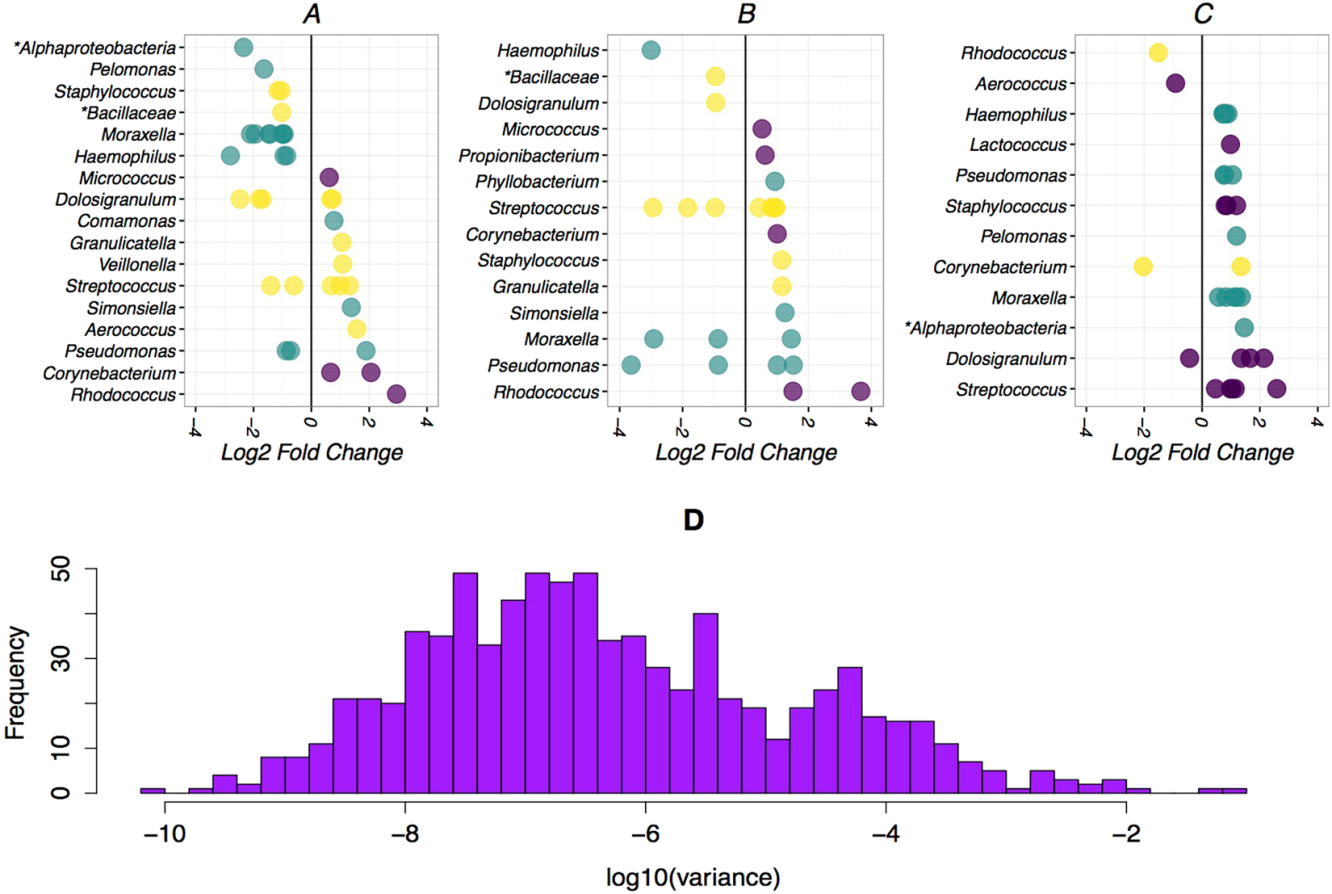
Differentially abundant OTUs across vaccination group in the first year of life. Differential abundance of individual OTUs across the vaccination groups was evaluated by the use of the negative binomial distribution and shrinkage estimator for the distribution’s variance using the DESeq. 2 extension in the Phyloseq package in R. OTUs that were differentially abundant (alpha <0.01) between Group 1 and 2 (**A**), Group 1 and 3 (**B**), and Group 2 and 3 (**C**). The log10 variance of most OTUs across the samples was very low (**D**). The colours represent the phylum to which the OTUs belong: purple circles represent *Firmicutes*, green circles represent *Proteobacteria* and yellow circles represent *Actinobacteria*. The asterisk (*) indicates a taxanomic Class while the other labels represent Genera.

Differential abundance of individual OTUs across the vaccination groups was evaluated by the use of the negative binomial distribution and shrinkage estimator for the distribution’s variance. The variance of most OTUs across all the nasopharyngeal samples was very low (<log10 (−4) as shown in Fig. 6. There were 38, 27, and 33 OTUs that had differential abundance between groups 1 & 2, groups 1 & 3 and groups 2 & 3 respectively (Fig. 6A,B and C). The OTUs with differential abundance across all the groups represented 56 unique OTUs belonging to the Firmicutes, Proteobacteria and Actinobacteria phyla. A total of 9 OTUs representing *Streptococcus*, *Moraxella*, *Dolosigranulum*, *Haemophilus*, *Pseudomonas* and *Pelomonas* genera had significantly different abundance across the vaccination groups after controlling for confounders in a mixed effect model performed as described above (Table 2). Group 2 and Group 3 infants had reduced abundance of 7 of the 9 OTUs compared to Group 1 infants.

**Table 2:** OTUs with differential abundance by vaccination group in the first year of life.

OTU	Taxanomic classification (Phylum; Class; Order; Family; Genus	Group	Frequency(%) (95% CI)	Mean Abundance% (Range)	Coef. (95% Cl)	*P-value*
*169*	*Proteobacteria*; *Gammaproteobacteria*; *Pseudomonadales*; *Pseudomonadaceae*, *Pseudomonas*	1	43 (39–48)	0.41 (0–24.78)	0.00	—
		2	18 (15–22)	0.01 (0–0.55)	−0.77 (−1.34–−0.21)	0.01
		3	30 (26–34)	0.02 (0–0.43)	−1.01 (−1.51–−0.51)	0.00
*2*	*Firmicutes*; *Bacilli*; *Lactobacillales*; *Streptococcaceae*, *Streptococcus*	1	99 (98–100)	29.74 (0–100)	0.00	
		2	99 (98–100)	32.09 (0–95.74)	0.14 (−0.15–0.42)	0.35
		3	99 (98–100)	35.82 (0–100)	0.46 (0.19–0.74)	<0.01
*203*	*Firmicutes*; *Bacilli*; *Lactobacillales*; *Streptococcaceae*; *Streptococcus*	1	46 (42–51)	0.21 (0–3.25)	0.00	—
		2	25 (21–29)	0.01 (0–0.32)	−0.71 (−1.32–−0.11)	0.02
		3	37 (33–41)	0.02 (0–0.97)	−0.78 (−1.35–−0.21)	0.01
*205*	*Proteobacteria*; *Gammaproteobacteria*; *Pseudomonadales*; *Moraxellaceae*; *Moraxella*	1	4 (35–44)	0.46 (0–40.47)	0.00	—
		2	27 (23–31)	0.07 (0–3.36)	−0.36 (−1.07–0.35)	0.32
		3	33 (29–37)	0.03 (0–2.34)	−0.95 (−1.6–−0.3)	<0.01
*3*	*Proteobacteria*; *Gammaproteobacteria*; *Pseudomonadales*; *Moraxellaceae*; *Moraxella*	1	91 (88–93)	6.32 (0–69.94)	0.00	—
		2	86 (83–89)	5.49 (0–72.88)	0.21(−0.18–0.61)	0.29
		3	94 (92–96)	7.32 (0–79.12)	0.57 (0.19–0.95)	<0.01
*46*	*Firmicutes*; *Bacilli*; *Lactobacillales*; *Carnobacteriaceae*; *Dolosigranulum*	1	73 (69–78)	1.65 (0–26.37)	0.00	—
		2	62 (57–67)	0.42 (0–9.78)	−1.02 (−1.67–−0.38)	<0.01
		3	74 (7–77)	0.84 (0–21.34)	−0.94 (−1.55–−0.33)	<0.01
*48*	*Proteobacteria*; *Betaproteobacteria*; *Burkholderiales*; *Comamonadaceae*; *Pelomonas*	1	34 (29–38)	0.18 (0–6.89)	0.00	—
		2	23 (19–27)	0.09 (0–18.01)	−0.54(−1.08–0.01)	0.05
		3	34 (31–38)	0.1 (0–3.16)	−0.58 (−1.08–−0.08)	0.02
*8*	*Proteobacteria*; *Gammaproteobacteria*; *Pasteurellales*; *Pasteurellaceae*; *Haemophilus*	1	77 (73–81)	4.44 (0–86.33)	0.00	—
		2	74 (7–78)	4.4 (0–74.06)	0.66 (0.19–1.12)	0.01
		3	85 (82–88)	5.74 (0–80.8)	0.64 (0.19–1.08)	0.01
*89*	*Proteobacteria*; *Gammaproteobacteria*; *Pasteurellales*; *Pasteurellaceae*; *Haemophilus*	1	33 (29–37)	0.45 (0–23.73)	0	—
		2	26 (22–3)	0.03 (0–1.37)	−0.27 (−0.91–0.38)	42
		3	25 (22–29)	0.02 (0–0.92)	−0.85 (−1.47–−0.23)	0.01

The mixed effect random intercept model was adjusted for time-invariant explanatory variables (gender, place of birth, number of siblings, maternal age) and time-variant explanatory explanatoryy (time, vaccination period, season, breast feeding, travelling, antibiotic usage and nutritional status based on weight for height).

## Discussion

There is growing concern that vaccination and antimicrobial treatment strategies that alter the normal balance of microbial communities could have temporal or pervasive adverse effects on health^20^. In this unique study of infants followed up from birth to 1 year with intensive sampling, we have demonstrated that while PCV7 vaccination effectively reduces the nasopharyngeal carriage of vaccine serotypes, pneumococcal carriage remains high (above 75%) among vaccinated infants due to an immediate expansion of non-vaccine serotypes throughout the first year of life. We have concurrently shown that the overall nasopharyngeal microbial community composition and structure remain largely unaltered by exposure to PCV7, even in the presence of herd immunity. These findings together suggest that exposure to PCV7 does not significantly alter the structure and composition of the infant nasopharyngeal microbiome among Gambian infants, likely due to immediate replacement of vaccine by non-vaccine serotypes.

Replacement of vaccine with non-vaccine serotypes associated with PCVs was reported even before the vaccine was first licensed. Following a small study of an experimental pentavelent CRM_197_ PCV among children, Obaro and colleagues reported a reduction in the carriage of vaccine types, which was countered by a commensurate increase in the carriage of non-vaccine serotypes^21^. Virtually complete replacement of vaccine with non-vaccine serotypes in carriage associated with PCV vaccination has been reported in several randomized controlled trials in South Africa^22^, the Netherlands^23^, Israel^24^, the United States of America^25^ and subsequently in The Gambia^26^. A new insight this study adds is how rapidly this replacement takes place among infants. Nasopharyngeal swabs were collected every two weeks which provided a unique opportunity to show that not only does clearance of vaccine serotypes occur rapidly (<14 days) following the first dose of PCV7 but serotype replacement also occurs concurrently (Fig. 2). This is in contrast to a previous report that suggested that reductions in vaccine-serotype carriage occur within one or two months^27^.

The most common non-vaccine serotypes carried by vaccinated children were serotypes19A, 35B and 15B. Increases in carriage of these serotypes following PCV vaccination have been widely reported across the globe and previously reviewed^9^. Previous pneumococcal surveys conducted in the same region as this study show that all three serotypes were circulating in the population prior to widespread use of PCV7 in the Gambia^17, 28, 29^. A pre-PCV longitudinal study of pneumococcal carriage among Gambian infants showed that serotype 35B was amongst the most commonly isolated serotypes at first acquisition and was amongst the predominant serotypes, accounting for just below 4% of pneumococcal isolates^17^. Both unmasking and increased acquisition are the most probable drivers of the expansion of the non-vaccine serotypes following PCV7 vaccination^30^. The detection of multiple serotypes was enhanced by the use of the sweep method as opposed to the traditional method in which one or two colonies per specimen are picked for serotyping. However, it is important to note that the culture-based detection of pneumococci employed in this study has lower sensitivity than molecular methods^31, 32^. Molecular pneumococcal detection and serotyping tools have the added benefit of enhancing multiple serotype detection and providing quantitative data^19, 32, 33^.

Rapid replacement of vaccine with non-vaccine serotype pneumococci is likely to have important implications for invasive disease. Population-based surveillance in the Upper River Region of The Gambia conducted between May 2008 and December 2014 showed a 47% increase in non-vaccine-type invasive pneumococcal disease, which was coupled with a significant decline in vaccine-type invasive pneumococcal disease following the introduction of PCV7 and PCV13^4^. Interestingly, serotypes 19A and 35B were amongst the leading non-PCV7 serotypes causing invasive pneumococcal disease in The Gambia after the introduction of PCV7. It was also striking that 19A invasive pneumococcal disease persisted between 2013 and 2014 among children less than 5 years old despite widespread use of PCV13 in the country^4^.

At least one other study has investigated the impact of PCV7 on the nasopharyngeal microbiome. Biesbroek and colleagues investigated the impact of PCV7 vaccination on the nasopharyngeal microbiota among 97 vaccinated infants and 103 unvaccinated infants in the Netherlands^34^. The vaccinated infants received 3 doses of PCV7 at 2, 4 and 11 months of age. Deep sequencing of nasopharyngeal specimens collected at 12 and 24 months showed changes in the composition and diversity of the microbial communities associated with PCV7 vaccination. However, these changes were temporary. Of note were the increases in the abundance of *Veillonella*, *Prevotella*, *Fusobacterium*, *Streptococcus* and *Neisseria* observed at 12 months. In this study, we found at least 56 OTUs from the Firmicutes, Proteobacteria and Actinobacteria phyla that were differentially abundant across the vaccination groups. This included members of the *Streptococcus*, *Staphylococcus* and *Moraxella* species. After adjusting for possible confounders such as age, sex, season, birthplace, antibiotic treatment, travel history and malnutrition, only 9 OTUs had differential abundance across the vaccination groups (Table 2). Despite these differences, the overall microbiome structure and composition were indistinguishable across the vaccination groups throughout the first year of life. This is consistent with findings from a small study conducted among 60 children aged 12–59 months in Kenya in which the 10-valent pneumococcal non-typeable *Haemophilus influenzae* protein-D conjugate vaccine (PHiD-CV) was not found to significantly alter the nasopharyngeal microbiome^35^.

Although a few studies have reported changes in the carriage of other pathogens such as *Staphylococcus aureus* and *Haemophilus influenzae* that co-colonise the nasopharynx following PCV vaccination^36–38^, it is not clear if there is a link with nasopharyngeal carriage dynamics. However, this does not rule out the possibility that localised changes in microbial ecology at the sites of infection, such as the middle ear in AOM, may play an important role^39–43^. It may be of some interest that the three OTUs that had significantly higher abundance among infants who received PCV7 and came from heavily vaccinated communities belonged to the *Haemophilus*, *Moraxella* and *Streptococcus* genera. This may warrant further investigation.

A key strength of this study is that the infants were intensively sampled in the first 6 months and followed up at regular intervals up to one year. A trade-off to the regular sampling was that the total number of infants recruited was only 102. Hence, more subtle changes in the nasopharyngeal microbiome composition and structure could have been missed. Group 1 was the control (unvaccinated) group in the initial study design, however, infants recruited into this group received PCV7 once it was introduced in The Gambia in August 2009. PCV7 was replaced by PCV13 in 2011 in The Gambia and it is unclear if the higher valency formulations will have similar impact on the nasopharyngeal microbiome.

The evidence presented here strongly suggests that as vaccine serotypes are cleared from the nasopharyngeal ecological niche, they are rapidly replaced by non-vaccine pneumococcal serotypes and not other bacterial species. Although this may provide some reassurance, diligent surveillance of nasopharyngeal carriage is crucial to ensure that the net public health gains of PCVs are maintained over the long term.

## Materials/Subjects and Methods

### Ethical permission

Ethical approval to conduct this prospective longitudinal study was granted by the Gambian Government and Medical Research Council Unit The Gambia Joint Ethics Committee. Written, informed consent was obtained from the participants’ parents or guardian at recruitment. Enrolment of participants and all procedures were conducted in accordance with the relevant regulations and guidelines.

### Study population

The Western Region is representative of rural areas in The Gambia. HIV prevalence is estimated at 2% and the majority of villagers belong to the Jola, Mandinka and Fula ethnic groups^29^. Villages in the Western Region, The Gambia covering an area of approximately 90 km^2^ were selected for this study. Study participants were recruited from 27 villages each with estimated birth rates between three and twenty-six per year. The villages were split into 3 groups of 9 villages with estimated population sizes of 2000 and birth rates of approximately 80 per year. Trained village reporters in each village recorded and reported pregnancies, births, deaths and other serious events. Recruitment of subjects was carried out between November 2008 and April 2009 after written informed consent. To avert recruitment bias, all newborns from participating villages for whom informed parental consent was obtained were recruited into the study until each of the vaccination groups had at least 30 children.

### Study vaccination groups

The study participants were recruited into three groups as outlined in the flowchart in Fig. 1. Group 1 and 2 participants were born in PCV7 unvaccinated villages while Group 3 participants were born in heavily vaccinated communities following the PCV7 vaccine trials conducted in 2006^2^. Group 2 and 3 infants received three doses of PCV7 at 2, 3 and 4 months whereas Group 1 infants did not receive PCV7 at this age. PCV7 was introduced in the Gambia in August 2009, 11 months after the study was started. Group 1infants received at least 1 dose of PCV7 during the national catch-up campaigns. All Group 1 infants were at least 6 months old when they received the first dose of PCV. Group 2 and Group 3 infants also received additional doses of PCV7 following the implementation of PCV7 in The Gambia after 6 months of age. Nasopharyngeal swabs were collected within seven days of birth, then biweekly for the first six months and subsequently bi-monthly up to one year from all participants.

### Nasopharyngeal sampling process

Nasopharyngeal swabs were collected as previously described^29^ by trained field workers. The swab was immediately inoculated into a vial containing 1 mL of chilled skim milk–tryptone–glucose-glycerol (STGG) transport medium. The vials were kept on ice, transported to the MRCG Laboratories site in Fajara and stored at −70 °C within eight hours of collection. Pneumococci were isolated as previously described^29^.

### DNA isolation for 454-pyrosequencing

Nasopharyngeal swabs stored in STGG were thawed on ice and gently vortexed for five seconds. DNA was extracted from each swab using the PowerSoil^®^ DNA Isolation Kit (MO BIO Laboratories, Carlsbad, CA, USA). 250 µL of the swab solution was transferred to the PowerBead® tubes and DNA was extracted following manufacture’s protocol. DNA was eluted in 100 µL of the kit elution buffer and immediately stored at −20 °C. DNA extractions were carried out in batches of 24 including one extraction control to which 250 µL of sterile DNAse free water was added instead.

### 16S rDNA sequencing

The V1-V3 region of the 16S rRNA gene were amplified using primers 5′-AGAGTTTGATCCTGGCTCAG-3′ and 5′*-*ATTACCGCGGCTGCTGG-3′ as previously described^44, 45^. The primers contained an adapter sequence and unique barcodes. The positive controls for all runs were purified. *Anaerotruncus colhominis* DNA, reagent controls and non-template controls were set up for each PCR run. Following amplification and purification, the amplicons were pooled at equimolar concentrations and sequenced on the 454 GS FLX Titanium Sequencing Platform (Roche, USA) as described elsewhere^44, 45^ at the Genome Institute (University of Washington in St. Louis, MO, USA).

Quality control and data processing were performed using in-house pipelines at the Jackson Laboratory. Briefly, reads with length <200 bp and/or with more than a single ambiguous base call were discarded. Chimeric sequences and reads without the adapter sequences were also removed. Reads from the same samples were binned based on barcode and then the barcode, adapter and primer sequences at both terminals were trimmed. Samples with read depth less than 500 were discarded based on rarefaction analysis of all the samples. Alignment and taxonomic classification (Phylum to Genus) of the reads was carried out with the Ribosomal Database Project Naïve Bayesian Classifier using a 0.5 filter.

### Data collection

At recruitment, background data including birth weight, birthplace, maternal age, maternal parity, ethnicity, sex, and vaccination status of mothers and siblings were collected. Socio-demographic and clinical information about the participants was collected at each visit including antibiotic use, anthropometric measurements, travel history, breastfeeding status, and infections (ear and chest). All data were collected on approved study forms and double entered and verified in an OpenClinica Electronic Data Capture system (Waltham, MA, USA) with an Access (Microsoft, Seattle, WA, USA) backend.

### Analysis

Differences in baseline characteristics of the study participants recruited into the three study groups were tested using the Chi square test, Fisher’s exact test or Kruskal-Wallis test where appropriate. A mixed effects model was used to compare richness and Shannon α diversity across the study groups. Richness was positively skewed and was square root transformed for statistical analysis. Time-invariant risk factors (gender, place of birth, number of siblings, maternal age) and time-variant risk factors (age, vaccination period, season, breast feeding, travelling, antibiotic usage and nutritional status based on weight for height) were used as explanatory variables. Age and vaccination group were the main exposure risk factors and the remaining explanatory risk factors were considered as potential confounders. A random-intercept model was used to handle these two types of explanatory risk factors. All explanatory risk factors mentioned above were included in the fixed effects component of the model as well as pairwise and triple interaction terms between time, group and period. A backward model selection approach using the Wald test was adopted. Explanatory risk factors with a non-significant effect at the 5% significance level were discarded. Model residuals and random effects were checked for reliability. Kaplan Meier survival curves were used to compare the rates to first acquisition of pneumococcus and PCV7 serotypes in the three study groups. Statistical analyses were performed using STATA/SE 14.1, USA. Microbial community composition, structure and ecology analyses were performed using the Phyloseq package^46^ with R version 3.2.1. To reduce noise, low-occurrence, poorly represented OTUs were filtered out i.e. OTUs that did not appear more than once in 10% of the samples. To evaluate the effect of PCV7 vaccination group on overall microbial composition, the phylogenetic dis(similarities) among communities from the three groups were evaluated by metric multidimensional scaling (MDS)/ Principle component analysis (PCoA) on weighted UniFrac distance. Differential abundance of individual OTUs across the vaccination groups was explored using the DESeq package-extensions in the Phyloseq package.

### Data availability

The datasets generated during and/or analysed during the current study are available from the corresponding author on reasonable request.

## Electronic supplementary material

Supplementary Information
