# Supplementary material for: Rapid replacement by non-vaccine pneumococcal serotypes may mitigate the impact of the pneumococcal conjugate vaccine on nasopharyngeal bacterial ecology
Source: Sci Rep. 2017 Aug 15;7:8127. doi: 10.1038/s41598-017-08717-0 (PMC5557800; doi:10.1038/s41598-017-08717-0)
Supplement: Supplementary file 1 — Supplementary Information [file 41598_2017_8717_MOESM1_ESM.pdf]

Rapid replacement by non-vaccine pneumococcal serotypes may mitigate the impact of the pneumococcal conjugate vaccine on nasopharyngeal bacterial ecology

Brenda Kwambana-Adams[1,3], Blake Hanson[2], Archibald Worwui[1], Schadrac Agbla[1,3], Ebenezer Foster-Nyarko[1], Fatima Ceesay[1], Chinelo Ebruke[1,3], Uzochukwu Egere[1], Yanjiao Zhou[2], Maze Ndukum[4], Erica Sodergren[2], Michael Barer[5], Richard Adegbola[6], George Weinstock[2] & Martin Antonio[1,3,7]

Supplementary Table 1. Mixed effects model investigating factors associated with richness

Variable	Category	Change in mean square root of richness (95% CI)	p	Overall p
**Age**	Age(weeks)	-0.01 (-0.03, 0.01)	0.30	0.30
**Group**	Group 1			
	Group 2	0.20 (-0.39, 0.78)	0.51	0.15
	Group 3	0.54 (-0.01, 1.09)	0.05	
**Period**	0 - 9 weeks			
	11-17 weeks	0.75 (0.41, 1.10)	<0.01	<0.01
	19 - 27 weeks	0.82 (0.30, 1.34)	<0.01	
	29 - 52 weeks	0.78 (-0.24, 1.79)	0.13	
**Siblings**	0			
	1-3	0.81 (0.01, 1.61)	0.048	0.04
	>3	1.25 (0.28, 2.22)	0.01	
**Mother's age**	<20			
	20-34	-1.19 (-2.08, -0.30)	<0.01	<0.01
	≥35 and above	-2.20 (-3.33, -1.07)	<0.01	
**Breastfeeding**	0			
	1	0.05 (-0.38, 0.48)	0.82	0.05
	2	-0.45 (-0.83, -0.07)	0.02	
	3	-2.04 (-4.68, 0.59)	0.13	

Supplementary Table 2. Mixed effects model investigating factors associated with Shannon diversity

Variable	Category	Change in Shannon diversity (95% CI)	p	Overall p
**Age**	Age(weeks)	-0.06 (-0.08, -0.03)	<0.01	<0.01
**Group**	Group 1			0.48
	Group 2	-0.06 (-0.21, 0.08)	0.40	
	Group 3	-0.09 (-0.23, 0.05)	0.22	
***Period**	0 - 9 weeks			0.43
	11-17 weeks	-0.21 (-0.71, 0.28)	0.40	
	19 - 27 weeks	-0.40 (-0.98, 0.18)	0.18	
	29 - 52 weeks	0.16 (-0.41, 0.73)	0.58	
**Travel**	No			0.02
	Yes	0.11 (0.01, 0.21)	0.02	
**Mother's age**	<20			0.02
	20-34	-0.15 (-0.35, 0.06)	0.18	
	35 and above	-0.29 (-0.51, -0.07)	0.01	
**Breastfeeding**	0			0.04
	1	-0.02 (-0.19, 0.14)	0.78	
	2	-0.20 (-0.34, -0.05)	<0.01	
	3	-0.70 (-1.70, 0.31)	0.18	
